# Use of Transient Time Response as a Measure to Characterize Phononic Crystal Sensors

**DOI:** 10.3390/s18113618

**Published:** 2018-10-25

**Authors:** Simón Villa-Arango, David Betancur, Róbinson Torres, Panayiotis Kyriacou

**Affiliations:** 1 Biomedical Engineering Research Group (GIBEC), EIA University, Envigado 055428, Colombia; david.betancur8@eia.edu.co (D.B.); robinson.torres@eia.edu.co (R.T.); 2 Research Centre for Biomedical Engineering (RCBE), City University of London, London EC1V 0HB, UK; p.kyriacou@city.ac.uk

**Keywords:** transient response, phononic crystal sensors, resonant sensors

## Abstract

Phononic crystals are periodic composite structures with specific resonant features that are gaining popularity in the field as liquid sensors. The introduction of a structural defect in an otherwise periodic regular arrangement can generate a resonant mode, also called defect mode, inside the characteristic band gaps of phononic crystals. The morphology, as well as the frequency in which these defect modes appear, can give useful information on the composition and properties of an analyte. Currently, only gain and frequency measurements are performed using phononic crystal sensors. Other measurements like the transient response have been implemented in resonant sensors such as quartz microbalances showing great results and proving to be a great complimentary measure to the gain and frequency measurements. In the present paper, a study of the feasibility of using the transient response as a measure to acquire additional information about the analyte is presented. Theoretical studies using the transmission line model were realized to show the impact of variations in the concentration of an analyte, in this case, lithium carbonate solutions, in the transient time of the system. Experimental realizations were also performed showing that the proposed measurement scheme presents significant changes in the resulting data, indicating the potential use of this measure in phononic crystal sensors. This proposed measure could be implemented as a stand-alone measure or as a compliment to current sensing modalities.

## 1. Introduction

Point-of-care (PoC) testing technologies are becoming an important area of development in the healthcare sector. When implemented correctly, PoC testing technologies can have a positive impact on operational efficiency and patient care [1].

PoC measurements provide results quickly, anywhere, and usually save a lot of time and costs. When PoC tests are performed, the samples are not required to be taken to a laboratory or healthcare facility to be analyzed with sophisticated equipment; they can, instead, be analyzed by a small, compact, and portable device that can deliver the results of the test immediately. With this type of technology, time-saving is almost assured; however, special care must be taken to avoid sacrificing quality or accuracy in the measurements [2].

Nowadays, acoustical, optical, and electrochemical technologies are positioning themselves as some of the most promising biomedical sensor technologies to develop new PoC applications. The miniaturization of conventional measurement techniques allows the realization of complex analytical systems [3].

Over the last years, a new type of resonant acoustic technology, phononic crystals, has been studied for biomedical sensing applications. Elastic and acoustic waves traveling through these structures are specifically modulated to generate frequency gaps that can be used to develop different applications [4]. 

Band gaps are produced mainly due to the effect of the periodicity created by spatial modulation of the acoustic impedance of the materials that compose the phononic crystal. The main properties that need to be taken into account when designing phononic crystal band gaps are the geometry, the mass density, and the speed of sound of the materials [4,5,6].

Phononic crystals can be fabricated using solids and fluids, the advantage of designing a structure that mixes both solids and fluids is that the acoustic impedance mismatch between the materials is very large, thus, resulting in broader and deeper bandgaps [7,8,9,10].

1D phononic crystals, also called multilayered phononic crystals or superlattices are formed by a series of thin layers with a significant impedance mismatch between consecutive layers and with large lateral dimensions compared to their thickness. This type of layer arrangement facilitates the selective reflection of the waves and the generation of bandgaps and has been previously used to develop sensing applications [11,12,13,14,15].

In order to be able to use phononic crystals as sensors, specific transmission features need to be introduced into the frequency response of the system. Defect modes can be utilized for this purpose. A defect mode is a resonant mode introduced by disrupting the symmetry of an otherwise periodic regular structure. The geometry and material properties of the defect need to be carefully selected so that a transmission band appears inside the band gap. Defect modes are engineered so that the material composing them is the analyte. Therefore, variations in the analyte composition result in changes in the transmission band resulting from the defect introduction [16,17]. 

Different sensing applications have been proposed using 1D phononic crystals. Some of the current measures used to characterize phononic crystal sensors are the frequency of maximum transmission, the peak amplitude, and the peak bandwidth [11,12,13,14,15,16,17].

Various studies have been presented on alternative measures that are of great use in other resonant sensors like the quartz crystal microbalance. One of the most promising alternative measurement modalities that has not been yet been proven in phononic crystals is transient time, especially when sensing liquids [18,19].

The present article introduces a new type of measurement for phononic crystals that uses the transient response to detect changes in the properties of an analyte.

## 2. Materials and Methods 

### 2.1. Phononic Crystal Sensor

**Figure 1:**
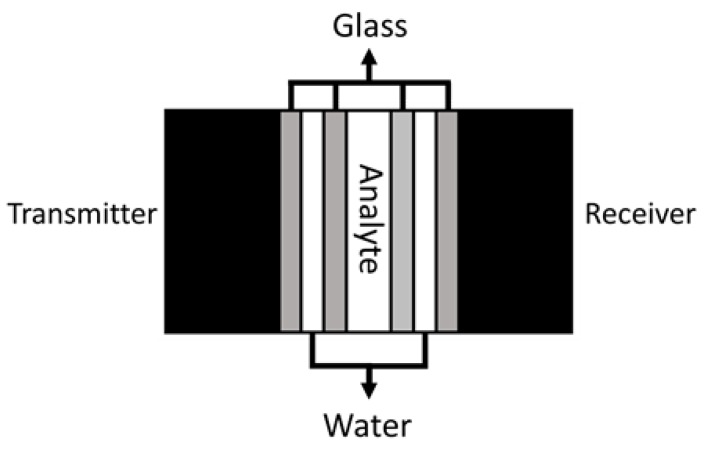
Phononic crystal structure representation.

A multilayered phononic crystal sensor was used to evaluate the utilization of transient time as a measure to characterize phononic crystals. The structure of the phononic crystal is composed of nine layers, and the materials used to build it are glass, distilled water, and lithium carbonate solutions as the analyte. The properties of the layers are shown in Table 1. The generation and acquisition of the mechanical waves are performed using wide bandwidth contact ultrasonic transducers configured as transmitters and receivers as can be seen in Figure 1. These transducers are represented in Table 1 as the outer layers of the crystal and are made of piezoelectric material. The transducers are considered as having semi-infinite dimensions for the simulations and their resonant frequency is located around 1.1 MHz.

Lithium carbonate was selected as the analyte since it is a very interesting and challenging PoC application. Multiple approaches have been used to try and detect it in blood due to its use as a therapeutic means for multiple psychiatric disorders [20,21,22]. The lithium carbonate solutions are made using distilled water and have a very low concentration. Therefore, their longitudinal speed of sound is almost the same as that of the distilled water. The analyte layer has a layer thickness of two times of that of the water layers so that a defect mode can be introduced in between the bandgap borders.

**Figure 2:**
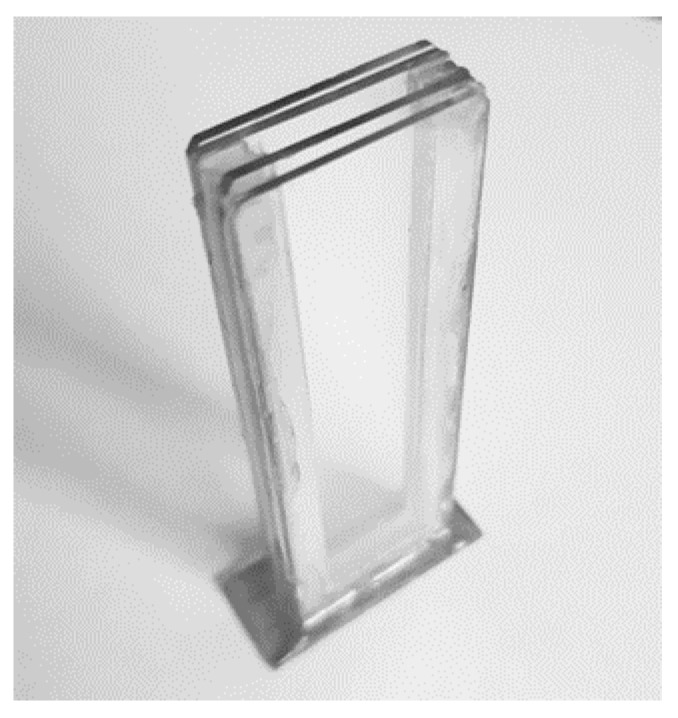
Phononic crystal sensor used for the experimental realizations.

**Figure 3:**
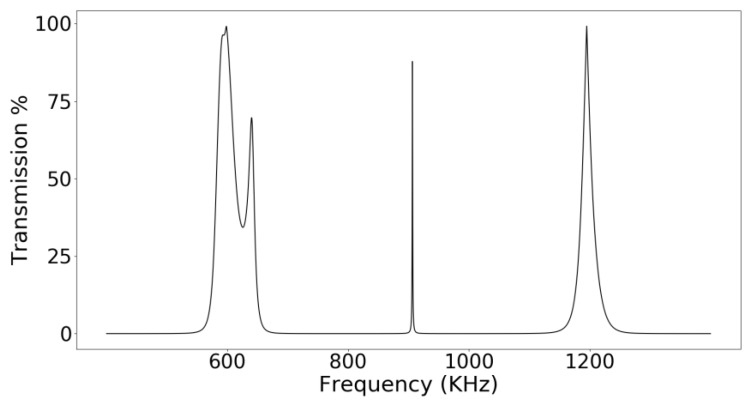
Simulated frequency response of the phononic crystal sensor.

Figure 2 shows the phononic crystal sensor used for the experimental realizations and Figure 3 shows the frequency response of the sensor obtained using the transmission line model. This simulation method uses a lateral miniaturization of the structure [23] and has been widely used to simulate resonant structures and phononic crystal sensors showing accurate results despite the use of a 1D model [14,17,24].

### 2.2. Signal Generation and Acquisition

A Tektronix oscilloscope (TDS 2012B), an AD8302 integrated circuit from Analog devices (Norwood, MA, USA), and a Tektronix precision signal generator (AFG 3101, Tektronix, Beaverton, OR, USA) were used to generate the signal and acquire the response of the system. All the instruments were connected to the computer via USB using an ATmega2560 microcontroller from Atmel (San Jose, CA, USA). A python code was written to control both the generation and the acquisition of the signal to synchronize the input and output and be able to obtain the transient response of the system.

### 2.3. Solutions Preparations

Lithium Carbonate (Li_2_CO_3_) solutions at different concentrations, Table 2, were made following the next protocol:

First, different containers were marked and separated depending on each concentration. Then, different amounts of Li_2_CO_3_ where weighted in a precision scale placing the lithium carbonate powder on top of an aluminum square. The different amounts needed for preparing the solutions were weighted, and finally, they were introduced in the containers together with the distilled water that was measured using a precision pipette. The different solutions were mixed until the solution was homogenized.

### 2.4. Protocol for the Tests

For the frequency and transient response tests, the following protocol was used. First, a syringe is used to introduce distilled water into every liquid layer of the phononic crystal shown in Figure 2. Then glycerol was applied as a coupling means between the ultrasonic transducers and the outer glass layers. Without a coupling agent, the signal is mostly attenuated due to the imperfect match of the layers and the air in between. Then connect the transmitter to the Tektronix generator and the receiver to the AD8302 integrated circuit together with a reference generator with a constant gain. Run the python program and watch if the signal is correct. When the sensor and instruments are fully operational, the analyte was carefully deposited in the central layer of the crystal. Each measurement was taken three times, and the phononic crystal was carefully cleaned and dried before each measurement. The tests were performed under a controlled room temperature since changes in temperature could affect the speed of sound of the materials and influence the results of the study.

### 2.5. Signal Processing

The signals were acquired and filtered by a second order low pass Butterworth filter with a cutoff frequency of 400 Hz to eliminate unwanted noise. A high-frequency value was selected as the cutoff frequency of the filter in order to avoid affecting the morphology of the resulting frequency spectra.

## 3. Results and Discussion

### 3.1. Frequency Sweep

**Figure 4:**
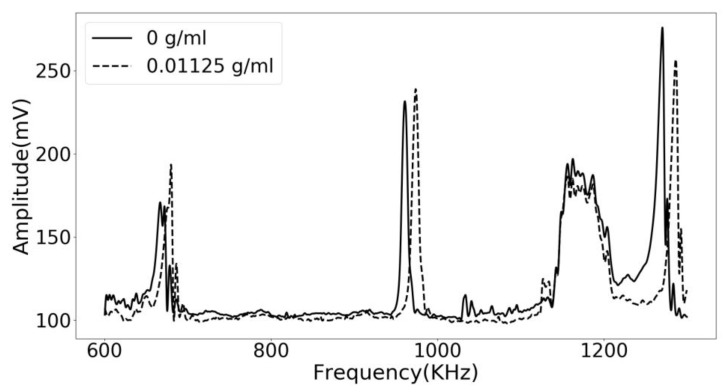
Experimental results of the phononic crystal sensor using distilled water (solid line) and a lithium carbonate solution with a concentration of 0.01125 g/mL (dashed line).

The operating frequency of the phononic crystal sensor as between 600 KHz and 1.3 MHz. As shown in Figure 4, running a frequency sweep along the phononic crystal resulted in a rejected band with a very distinct transmission band inside it. The transmission peak located around 1 MHz is generated by the defect mode introduced in the central layer of the phononic crystal. It is well separated from other transmission bands resulting in a significant signal to noise ratio. The solid line represents the resulting spectra using distilled water as the analyte. It is clear how adding a low concentration of lithium carbonate affected the resulting frequency spectra and made the defect mode displace itself to higher frequencies. The total displacement of the resonant mode is about 15 KHz.

**Figure 5:**
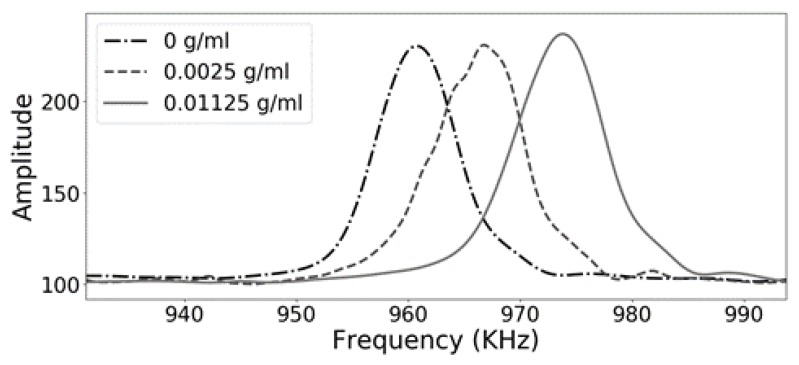
Zoomed-in experimental results of the phononic crystal sensor using distilled water (black dashed and dotted line), and lithium carbonate solutions with a concentration of 0.01125 g/mL (gray solid line) and 0.0025 g/mL (gray dashed line).

Figure 5 shows a zoomed-in view of the defect mode of the phononic crystal sensor. It is very interesting how changes in the concentration of the lithium carbonate can be tracked by measuring the frequency of this transmission features, showing the potential of using phononic crystals as sensors and for developing PoC applications.

### 3.2. Transient Response

**Figure 6:**
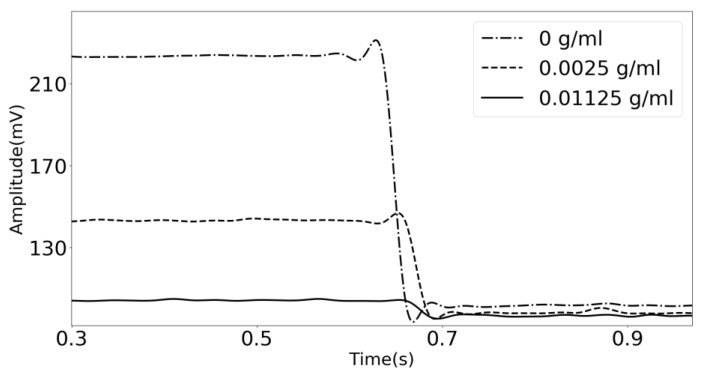
Transient time experimental results of the phononic crystal sensor using distilled water (dashed and dotted line), and lithium carbonate solutions with a concentration of 0.01125 g/mL (solid line) and 0.0025 g/mL (dashed line).

In Figure 6, the transient response of the acoustic waves traveling across the phononic crystal sensor at three different concentrations is shown. The system was set to a fixed frequency of 960 KHz, frequency of maximum transmission when using distilled water as the analyte, and the gain was recorded against the time. The generator system is suddenly turned off, and the transient response is obtained. The concentration-dependent damping observed in Figure 6, is related to the displacements in the frequency of the resonant transmission feature located in the middle of the bandgap. Because the amplitude of the starting signal is higher when distilled water is used as the analyte (0 g/mL), the transient response is more extended, and the slope of the signal is more inclined. When the lithium carbonate concentration increases, the initial amplitude is very low, resulting in a shorter decay time and a less steep slope. 

The same experimental realization was performed with the six lithium carbonate solutions that were previously prepared. The decay time of each signal was measured and is shown in Table 3.

**Figure 7:**
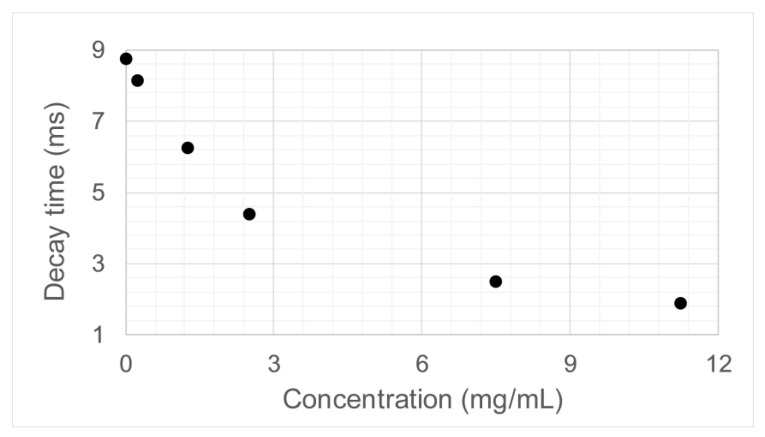
Relationship between the concentration of the lithium carbonate solution used as the analyte and the decay time obtained.

Figure 7 shows how the decay time changes when the concentration of the analyte is varied.The decay time values show a clear tendency and could be used as a measure to characterize the phononic crystal sensor and retrieve the concentration value of the analyte. 

## 4. Conclusions

A phononic crystal sensor that uses the transient response to quantify small changes in the properties of liquid analytes was developed. The sensor was tested using solutions of Lithium Carbonate in distilled water at different concentrations.

The use of a new variable to measure changes in properties of an analyte opens the door to an entirely new type of sensing modality that can complement the traditional gain and frequency measurements that are performed nowadays.

The transient response experimental realizations show that the slope and time decay vary when the concentration of the analyte is modified, allowing its use as a measure in sensing applications.

The experimental results show that the use of phononic crystal sensors for developing PoC applications for measuring lithium carbonate has a high potential. However, it is essential to take into account that the transmission feature frequency is sensitive to variations in the speed of sound of the sample. Blood is a complex mixture and, therefore, proper care needs to be taken to be able to make the system specific to changes in the concentration of lithium and avoid false readings due to changes in other elements of the mixture.

## Figures and Tables

**Table 1:** Properties of the layers.

Layer Number	Material	Speed of Sound (m/s)	Density (Kg/m^3^)	Thickness (mm)
1	PZT	3333	7500	-
2	Glass	2200	5720	1.25
3	Water	998	1493	1.25
4	Glass	2200	5720	1
5	Analyte			2.5
6	Glass	2200	5720	1
7	Water	998	1493	1.25
8	Glass	2200	5720	1.25
9	PZT	3333	7500	-

The layer thickness of the PZT layers is considered as semi-infinite for the simulation.

**Table 2:** Lithium carbonate solutions.

Solution #	Concentration (g/mL)
1	0
2	0.00025
3	0.00125
4	0.0025
5	0.0075
6	0.01125

**Table 3:** Decay time at different concentration values.

Concentration (g/mL)	Time (s)
0	0.008750
0.00025	0.008125
0.00125	0.006250
0.0025	0.004375
0.0075	0.002500
0.01125	0.001875
